# Supplementary material for: A UMLS-based spell checker for natural language processing in vaccine safety
Source: BMC Med Inform Decis Mak. 2007 Feb 12;7:3. doi: 10.1186/1472-6947-7-3 (PMC1805499; doi:10.1186/1472-6947-7-3)
Supplement: Additional file 13 — RAP application directory. Sets up directory for RAP (RDF application for PHP) source code [file 1472-6947-7-3-S13.gz › rap/doc/phpdoc/__filesource/fsource_syntax__syntaxGRDDLParser.php.html]

xml version="1.0" encoding="iso-8859-1"?


File Source for GRDDLParser.php


# Source for file GRDDLParser.php

Documentation is available at GRDDLParser.php

```
1. <?PHP
2. // ----------------------------------------------------------------------------------
3. // Class: RdfParser
4. // ----------------------------------------------------------------------------------


10. /**
11. * A GRDDLParser.
12. * This class extracts rdf data from xhtml documents. It uses the PHP xsltprocessor.
13. * Gleaning Resource Descriptions from Dialects of Languages (GRDDL):
14. * (http://www.w3.org/TR/grddl/)
15. *
16. * <BR><BR>History:<UL>
17. * <LI>10-26-2004                 : first version</LI>
18. * </UL>
19. *
20. * @version  V0.9.1
21. * @author Tobias Gau� <tobias.gauss@web.de>,
22. *
23. * @package syntax
24. * @access    public
25. *
26. */
27. class GRDDLParser extends Object{


30. /**
31. * Document link
32. *
33. *
34. * @var     String
35. * @access    private
36. */
37. var $doclink;


40. /**
41. * Stylesheet link
42. *
43. *
44. * @var     String[]
45. * @access    private
46. */
47. var $stylelinks;


50. /**
51. * DomDocument
52. *
53. * @var     DomDocument
54. * @access    private
55. */
56. var $domdoc;


59. /**
60. * generates a MemModel and creates the DomDocument.
61. *
62. * @param String $doc
63. * @access public
64. * @return MemModel $model
65. */
66. function generateModel($doc){
67. $model = new MemModel();
68. $this->doclink=$doc;
69. $this->domdoc = new DomDocument;
70. $this->domdoc->load($doc);
71. $this->_getStyles();
72. $model = $this->_generateRDF();
73. return $model;
74. }


77. /**
78. * gets the used xsl stylesheets.
79. *
80. * @access private
81. */
82. function _getStyles(){
83. $link=$this->domdoc->getElementsByTagName('link');
84. $i=0;
85. while($link->item($i)!=''){
86. $item = $link->item($i);
87. if($item->getAttributeNode('rel')->value=='transformation'){
88. $temp = $item->getAttributeNode('href')->value;
89. if(substr($temp,0,1)=='/'){
90. $pos = strrpos($this->doclink,'/');
91. $part = substr($this->doclink,0,$pos);
92. $this->stylelink[]=$part.$temp;
93. }else{
94. $this->stylelink[]=$temp;
95. }
96. }
97. $i++;
98. }
99. }

101. /*
102. * uses the PHP build in xslt processor to
103. * generate the RDF statements and uses the
104. * RDF- Parser to generate the model
105. *
106. * @access private
107. * @return MemModel $model
108. */
109. function _generateRDF(){
110. $model=new MemModel();
111. $model->setBaseURI($this->doclink);
112. $proc = new xsltprocessor;
113. include_once(RDFAPI_INCLUDE_DIR.PACKAGE_SYNTAX_RDF);
114. $pars=new RdfParser();
115. foreach($this->stylelink as $key => $value){
116. $xsl = new DomDocument;
117. $xsl->load($value);
118. $proc->importStyleSheet($xsl);
119. $model->addModel($pars->generateModel($proc->transformToXML($this->domdoc),$this->doclink));
120. }
121. return $model;
122. }


126. }
127. ?>
```

Documentation generated on Thu, 7 Jul 2005 13:41:28 +0200 by phpDocumentor 1.3.0RC3
